# Methylation of *MYLK3* gene promoter region: a biomarker to stratify surgical care in ovarian cancer in a multicentre study

**DOI:** 10.1038/bjc.2017.83

**Published:** 2017-03-28

**Authors:** David L Phelps, Jane V Borley, Kirsty J Flower, Roberto Dina, Silvia Darb-Esfahani, Ioana Braicu, Jalid Sehouli, Christina Fotopoulou, Charlotte S Wilhelm-Benartzi, Hani Gabra, Joseph Yazbek, Jayanta Chatterjee, Jacey Ip, Harun Khan, Marina-Therese Likos-Corbett, Robert Brown, Sadaf Ghaem-Maghami

**Affiliations:** 1 Department of Surgery and Cancer, Imperial College London, Hammersmith Campus, Du Cane Road, London W12 0NN, UK; 2 Institute of Pathology, Charité Universitätsmedizin, Berlin 10117, Germany; 3 Department of Gynaecology, Virchow Campus, Universitätsmedizin, Berlin, Germany

**Keywords:** ovarian cancer, surgery, biomarker, *MYLK3*, cg13247990, DNA methylation, CpG

## Abstract

**Background:**

Survival benefit from surgical debulking of ovarian cancer (OC) is well established, but some women, despite total macroscopic clearance of disease, still have poor prognosis. We aimed to identify biomarkers to predict benefit from conventional surgery.

**Methods:**

Clinical data from women debulked for high-stage OC were analysed (Hammersmith Hospital, London, UK; 2001–2014). Infinium’s HumanMethylation27 array interrogated tumour DNA for differentially methylated CpG sites, correlated to survival, in patients with the least residual disease (RD; Hammersmith Array). Validation was performed using bisulphite pyrosequencing (Charité Hospital, Berlin, Germany cohort) and The Cancer Genome Atlas’ (TCGA) methylation data set. Kaplan–Meier curves and Cox models tested survival.

**Results:**

Altogether 803 women with serous OC were studied. No RD was associated with significantly improved overall survival (OS; hazard ratio (HR) 1.25, 95% CI 1.06–1.47; *P=*0.0076) and progression-free survival (PFS; HR 1.23, 95% CI 1.05–1.43; *P=*0.012; Hammersmith database *n*=430). Differentially methylated loci within *FGF4*, *FGF21*, *MYLK2*, *MYLK3*, *MYL7*, and *ITGAE* associated with survival. Patients with the least RD had significantly better OS with higher methylation of *MYLK3* (Hammersmith (HR 0.51, 95% CI 0.31–0.84; *P=*0.01), Charité (HR 0.46, 95% CI 0.21–1.01; *P=*0.05), and TCGA (HR 0.64, 95% CI 0.44–0.93; *P=*0.02)).

**Conclusions:**

*MYLK3* methylation is associated with improved OS in patients with the least RD, which could potentially be used to determine response to surgery.

Ovarian cancer (OC) is the most lethal gynaecological malignancy, causing over 125 000 deaths per year worldwide (Sankaranarayanan and Ferlay, 2006). Overall survival (OS) is poor due to late presentation, poor surgical outcomes, and the development of chemotherapy resistance. The disease is clinically and molecularly heterogeneous but despite this all patients are treated the same, with cytoreductive surgery and platinum-based chemotherapy (NICE, 2011).

Surgical effort is one of the most important prognostic factors in OC (Agarwal and Kaye, 2003). It is widely accepted that size of residual disease (RD) following surgery influences OS following treatment (Pomel *et al*, 2008; Shih and Chi, 2010). Five-year OS has been shown to improve from 23.9 to 54.6 months in women having surgical resection to no macroscopic RD *vs* RD>10 mm (Wimberger, 2010). As surgical expertise and effort have improved total macroscopic clearance rates, it has become apparent that some patients still respond poorly to treatment, despite complete removal of all macroscopic tumour at primary surgery.

Gene expression signatures are associated with different survival outcomes in high-grade serous OC (HGSOC) patients (Tothill *et al*, 2008; TCGARN, 2011). Prognostic biomarkers associated with survival in HGSOC have been widely investigated in the literature (Fiegl *et al*, 2008; Flanagan *et al*, 2013). Notable examples include overexpression of individual genes such as *VEGF*, *HER2* and progesterone receptor, which have been associated with progression-free survival (PFS) and OS (Yu *et al*, 2013; Zhao *et al*, 2013). Differential DNA methylation at CpG sites, particularly at CpG islands in gene promoter regions, is also associated with PFS and OS of HGSOC patients (Dai *et al*, 2011, 2013; Wang *et al*, 2014; Chou *et al*, 2015). These findings improve our understanding of specific tumour biology and raise the possibility of utilising these biological characteristics to allow patient stratification to treatment. Differential methylation of *O*-6-methylguanine-DNA methyltransferase is already in use clinically to stratify treatment in those with malignant glioblastomas (Hegi *et al*, 2005; Malmstrom *et al*, 2012; Gupta *et al*, 2015).

To our knowledge there are no current biomarkers that can be used to identify women with HGSOC who do poorly despite optimal surgical treatment (Borley *et al*, 2012). Thus, there is a proportion of these patients who undergo maximal effort radical surgical treatment, with all of the associated potential morbidity and mortality, without the associated survival benefit. For women who have a poor prognosis, despite maximal surgical reduction in disease, alternative therapeutic approaches would need to be applied in a context of individualised surgical treatment.

The aim of this study was to identify the proportion of women that have poor prognosis despite maximal surgical effort. Also we aimed to identify and validate potential DNA methylation biomarkers, which may enable stratification of care to different surgical and treatment pathways. DNA methylation as a biomarker was selected due to its relative stability *ex vivo* and *in vivo*, as well as the future potential to detect DNA methylation changes non-invasively in circulating tumour DNA (Keeley *et al*, 2013; Bettegowda *et al*, 2014). As the success of surgical debulking is associated with the ability of tumour cells to invade and metastasise, we focused on the ‘focal-adhesion’ and ‘adherens-junction’ pathways, involved with cell surface adhesion, and the ‘regulation of actin cytoskeleton’ pathway involved with cell migration (Laboratories, 2013).

## Patients and methods

This proposal was reviewed and agreed by the ‘HTA-approved’ Imperial College Healthcare NHS Trust Tissue Bank (authorised by the Wales MREC) project reference Gyn_HG_12_060 and Gyn_HG_13_020. Written consent was obtained from all patients included in this study who provided tumour tissue for research. Reporting recommendations for tumour marker (REMARK) criteria were followed throughout this study.

### Historical Hammersmith database

Electronic patient records from January 2001 to December 2014 at Hammersmith Hospital, Imperial College Healthcare NHS Trust (ICHNT; West London Gynaecology Oncology Surgical Centre) were searched to identify cases. Inclusion criteria were primary serous ovarian, fallopian, or peritoneal carcinoma (mixed tumours were included if the epithelial component predominated), stage 3/4, and primary surgical debulk was initial treatment. Staging was classified using the version of International Federation of Gynaecology and Obstetrics staging classifications in place at the time of diagnosis. OS and PFS were determined from the date of surgery until the date of the event (death or recurrence/progression, respectively) or last known contact. All survival data were updated between 15 June 2015 and 03 July 2015. If patients were lost to follow-up they were censored accordingly. RD status was classified as total debulking (0 mm RD), optimal debulking (RD ⩽10 mm), and suboptimal debulking (>10 mm RD).

### Patient tumour samples

Fresh-frozen OC tissue was collected and stored at −80 °C by ICHNT Biobank London and Tumour Bank Ovarian Cancer (TOC) (www.toc-network.de) at Charité Hospital Berlin. Tumours were derived from primary debulking surgery and were stage 3/4 serous histology. Tumour sample quality was ensured by histopathology (excluded if tumour cell nuclei <20%). Overall survival and PFS were determined from the date of surgery until the date of the event (death or progression, respectively) or last known contact and censored if lost to follow-up. The Hammersmith and TOC-Charité DNA methylation data sets included serous tumour with similarly matched age and stage. Residual disease differed between the data sets, with suboptimal debulking rates lower in the TOC-Charité and The Cancer Genome Atlas (TCGA) data sets compared to Hammersmith. This may reflect the significant variability between surgeons’ approach to radicality, skills, and the significant difficulty in the reproducibility of tumour measurements, which corresponds with previously described variability among surgical expertise and outcomes among different centres and different countries (Chi *et al*, 2009; Vergote *et al*, 2010). The potential effect this may have on survival was mitigated by adjusting for RD as a confounder. Of all patients that were fit to have chemotherapy, over 95% received platinum-based chemotherapy.

### TCGA data set

Publicly available clinical, surgical, methylation, and expression data for patients with serous cystadenocarcinoma were downloaded from TCGA data portal (https://tcga-data.nci.nih.gov/tcga/; TCGARN, 2011). Methylation data were obtained using 27k and 450k Illumina Infinium (San Diego, CA, USA) methylation arrays. We included 277 patients with stage 3/4 disease. Supplementary Table S1 shows the clinical characteristics of the historical Hammersmith database cases and the three independent methylation data sets.

### Tumour DNA extraction and bisulphite conversion

Up to 500 mg of tumour tissue was used per sample, and DNA was extracted using the chlorinated Nucleon extraction (GE Healthcare Life Sciences, Buckinghamshire, UK) method. Tumour DNA was bisulphite-converted with EZ-96 DNA Methylation Kit (Zymo, Irvine, CA, USA) as per the manufacturer’s protocol. A unit of 1 μg of genomic DNA was used for each sample.

### Illumina Infinium HumanMethylation27 beadchip array

Discovery-set samples (Hammersmith array data set) were processed as per the Infinium Assay Methylation Protocol (Illumina). A unit of 200 ng of genomic DNA was used for bisulphite conversion. Methylation data were summarised as *β*-values, calculated as *M*/(*M*+*U*), where *M* is the signal from methylated beads and *U* is the signal from unmethylated beads at the targeted CpG site. *β*-values were adjusted against background noise and data were log-transformed to achieve a normal distribution. Probes with a detection *P*-value of >0.05 were removed. Quality control checks were performed with GenomeStudio (Illumina).

### Pyrosequencing

DNA methylation of the validation-set samples (TOC-Charité data set) was determined through PCR amplification with biotinylated primers (Invitrogen Life Sciences, Carlsbad, CA, USA; and Sigma Aldrich, St Louis, MO, USA) using Pyromark Assay Design Software version 2.0 (Supplementary Table S2), and Qiagen (Hilden, Germany) Pyromark Q96 MD pyrosequencer as previously described (Dai *et al*, 2013). Amplified products were confirmed with agarose gel electrophoresis. Pyro-Q-CpG (Qiagen) software was used to calculate CpG site methylation values using the same *M*/(*M*+*U*) calculation as with the beadchip array.

### Statistical analysis

Analysis was performed using R v3.2.2. Univariate analyses using the log-rank test were performed and corresponding log-rank *P*-values reported. The OS and PFS analyses were plotted using Kaplan–Meier survival curves. Patients lost to follow-up were censored and their last known contact used for survival purposes. Multivariable adjustment was performed for true confounders, including age, stage, grade, RD status, and batch using Cox proportional hazards models and shown as multivariable Cox *P*-values. Hazard ratios (HR) and 95% confidence intervals (95% CIs) are reported alongside the Cox *P*-values. Statistical significance was set at *P<*0.05.

## Results

### Changing surgical trends towards more radical surgery are associated with improved survival

**Figure 1:**
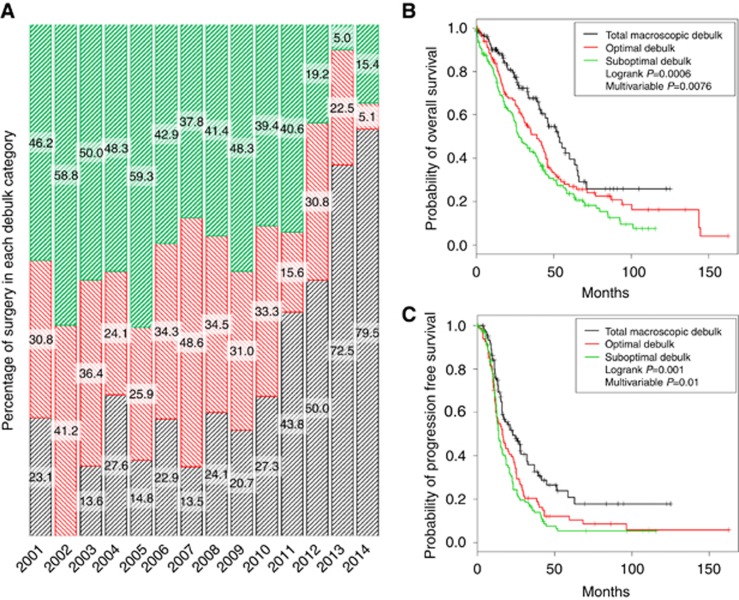
**Surgical trends and survival according to debulk status in the Hammersmith Database between 2001 and 2014.** (**A**) Debulk rates (percentage of all cases) at Hammersmith Hospital between 2001 and 2014. Total macroscopic debulk (0 mm RD) shown in black, optimal debulk (RD 1–10 mm) in red, and suboptimal debulk (RD>10 mm) in green. (**B**) Overall survival – total debulk *vs* suboptimal debulk (HR=1.25 (95% CI 1.06, 1.47)). (**C**) Progression-free survival – total debulk *vs* suboptimal debulk (HR=1.23 (95% CI 1.05, 1.43)). Multivariable Cox proportional hazards model adjusting for age, stage, grade, and histology.

Over the past 15 years, surgical trends have moved towards a more radical approach in response to mounting evidence of prognostic benefit with reduced tumour burden (Chi *et al*, 2009). To evaluate this trend at Hammersmith Hospital and to establish whether this impacted upon survival, debulking rates were compared year by year and survival of these patients was evaluated using Kaplan–Meier survival curves; Figure 1. In 2013 and 2014, less than a quarter of patients had any measurable disease left after surgery, compared with over 75% in 2001–2003 (Figure 1A). A total of 430 women who underwent primary debulking surgery were included in the analyses. The survival curves in Figure 1B and C show statistically significant OS (HR 1.25, 95% CI 1.04, 1.47) and PFS (HR 1.23, 95% CI 1.05, 1.43) advantage for women with the least RD after surgery. Median OS for patients with RD>10 mm was 13.2 months, compared to those with zero RD with median OS of 26.2 months. Median PFS (Figure 1C) improved with reducing RD as expected.

Despite patients receiving total macroscopic clearance, it is clear that some of these patients, even with maximal surgical effort, do poorly with respect to survival. Of all totally debulked (0 mm RD) patients in the Hammersmith database, 8.8% died within 12 months of their surgery. Median OS in this group of women was only 3 months (inter-quartile range 0.5–8.0 months). These patients may not have benefitted from extensive surgery and may have potentially benefitted from alternative treatment strategies, highlighting the need for a robust biomarker to identify these women. We therefore used three independent tumour DNA data sets to identify and validate DNA methylation biomarkers associated with survival in patients with the least RD.

### DNA methylation biomarkers predict poor survival in optimally debulked patients

To identify differential methylation associated with survival in optimally debulked patients, DNA methylation of tumour DNA determined through Illumina’s Infinium HumanMethylation27 BeadChip array was investigated (Hammersmith array data set). Multivariable Cox proportional hazards models were performed on optimally debulked patients only. The Kyoto Encyclopaedia of Genes and Genomes was used to identify 732 methylation probes covering genes involved with adhesion and migration pathways. Our hypothesis was that genes involved in this pathway could affect survival by increasing metastatic potential and tumour invasiveness, therefore leading to biologically more aggressive tumours. The hypothesis-driven analysis was performed on 732 loci and 65 were found to show differential methylation associated with OS (*P<*0.05). To increase the likelihood of detecting clinically relevant differential methylation we chose a differential methylation cut-off of 20%. Consequently, 36 probes were included in further analysis. Cox proportional hazards models were used to adjust for age, stage, chip, and RD to investigate whether patients had different survival distributions per locus. Of the 36 loci, 27 were found to be significant (Supplementary Table S3). The six probes with the largest effect size, *FGF4*, *FGF21*, *MYLK2*, myosin light chain kinase 3 (*MYLK3*), *MYL7*, and *ITGAE* are shown in Table 1, and were taken forward for validation.

### *MYLK3* CpG locus methylation predicts poor outcome in patients with the least RD

**Figure 2:**
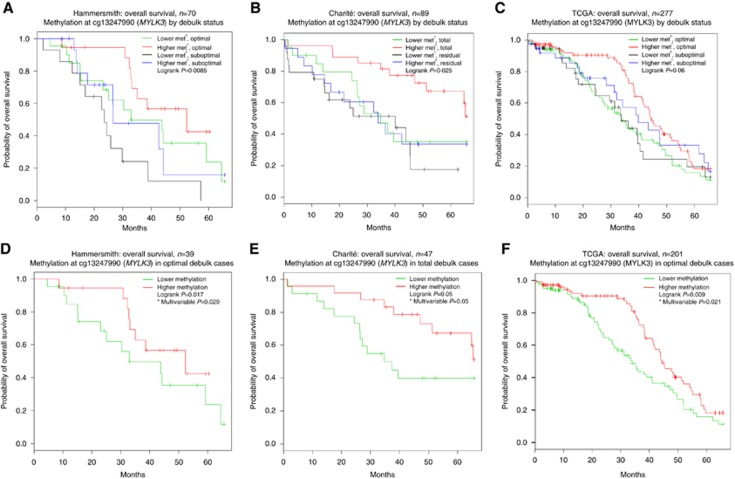
**Kaplan-Meier overall survival curves according to debulk status and methylation (met^n^), shown as higher or lower than the median methylation for all patients.** (**A**) Probe cg13247990 on 27k methylation array in discovery cohort (Hammersmith Array data set) with debulk status shown as ‘optimal’ *vs* ‘suboptimal’. (**B**) Pyrosequencing of *MYLK3* locus in validation cohort (Charité data set) with debulk status shown as ‘total’ debulk *vs* any ‘residual’ disease. (**C**) Probe cg13247990 on methylation array in TCGA data set with debulk shown as ‘optimal’ *vs* ‘suboptimal’. (**D**) Hammersmith patients with optimal debulk (HR=0.51 (95% CI 0.31, 0.84). (**E**) Charité patients with total debulk (HR=0.46 (95% CI 0.21, 1.01)). (**F**) TCGA patients with optimal debulk (HR=0.64 (95% CI 0.44, 0.93). *All multivariable analyses adjusted for confounders age and stage plus grade, chip, and RD status when appropriate.

Validation of the six significant loci associated with survival in the Hammersmith Array data set was performed using an independent data set (TOC-Charité). DNA methylation of all six loci in OC tumour DNA was quantified by bisulphite pyrosequencing of the TOC-Charité data set validation cohort. As there was a low number of suboptimally debulked patients in this group (*n*=6), total debulk (*n*=47) was compared to any amount of RD (*n*=36). *MYLK3* was the only locus to validate in TOC-Charité (*P*=0.025; Figure 2B). The same analysis was performed in TCGA data set for all six discovery loci (Supplementary Table S4). Although *MYLK3* did not quite reach significance (*P*=0.06), in the TCGA analysis there is a clear trend in the survival curves (Figure 2A–C) towards improved survival with increased methylation of *MYLK3* in all data sets. Survival for patients with low methylation appears to be comparable to that of their suboptimally debulked counterparts, who had significant RD. Patients appeared not to benefit from the radical surgery in the same way their highly methylated counterparts did.

Further multivariable analysis focused only on patients with the least RD, as clinically it would be beneficial to be able to predict which patients would benefit most from radical surgery. Differential methylation survival analysis of the *MYLK3* locus was conducted in the Hammersmith and TOC-Charité data sets, as well as TCGA data set (Table 2 and Supplementary Table S4). Higher methylation of the *MYLK3* locus is associated with OS in patients with the least RD in all three independent cohorts of patients (Figure 2D and E), however the TOC-Charité data set was borderline significant (*P*=0.053).

**Figure 3:**
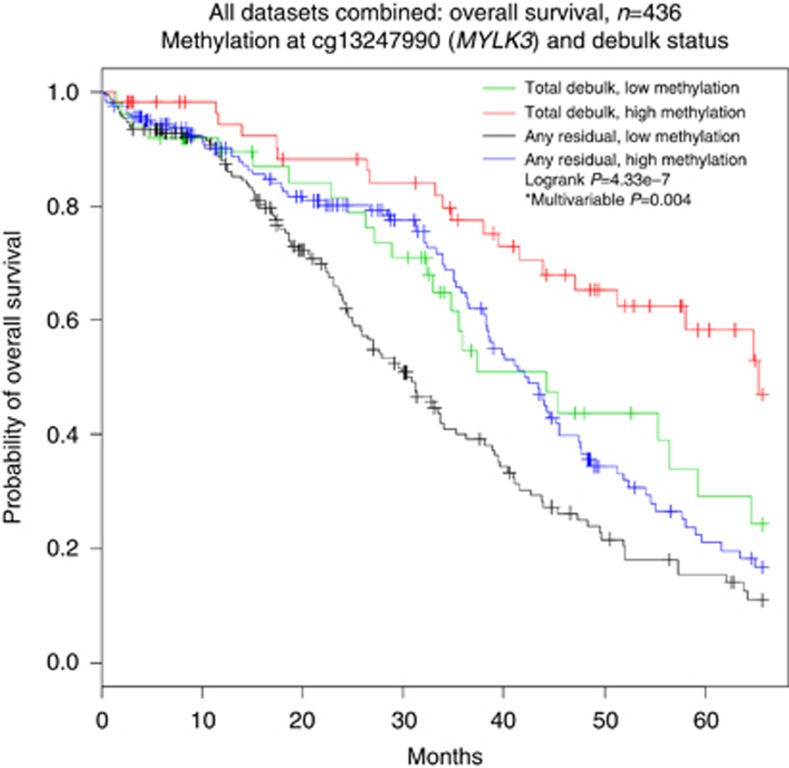
**Overall survival in all three data sets combined.** Overall survival significantly improved by reducing RD and increasing *MYLK3* methylation. Survival benefit from total debulk appears to be lost in the presence of low methylation of *MYLK3*. Similarly, women with RD gain survival from having high *MYLK3* methylation. Total of 436 women included from Hammersmith, Charité, and TCGA data sets. Survival dichotomised by median methylation of *MYLK3* derived from the total cohort (84.9%) and debulk status. *Multivariable model adjusted for age, stage, grade, debulk, batch, and an integer variable to adjust for combining data sets.

The optimally debulked Hammersmith patients with higher methylation at the *MYLK3* locus had median OS of 52.4 months, falling to 33.0 months in patients with lower methylation. Patients with higher methylation in TCGA data set had median OS of 44.5 months, a survival advantage of 10.5 months when compared to median OS of the low-methylation group, which was 34.0 months. Charité patients had the largest survival advantage from higher methylation in their totally debulked patients with median OS of 68.0 months with higher methylation, falling to 34.7 months in the lower-methylation group. On average, women with higher methylation of the *MYLK3* locus appear to have 10.5–33.2 months’ additional OS advantage across the three independent cohorts in this study. The TOC-Charité result may reflect that the effect of higher methylation at *MYLK3* is most profound in patients with total macroscopic clearance of their disease. To further investigate this theory, all three methylation data sets were combined, totalling 436 women for analysis in a similar manner to the TOC-Charité and TCGA validation analyses. The combination of all data sets increases the numbers of patients in all subgroups and enables an analysis of the entire cohort splitting the groups into ‘total macroscopic clearance of disease’ and ‘any RD’. Potential batch effects, combining these data sets, were adjusted for in the multivariable Cox model for this analysis as an indicator variable. Figure 3 shows a highly significant association (log-rank *P*=4.33e^−7^, Cox *P*=0.0040, HR 0.67, 95% CI 0.51, 0.88) between OS and methylation of *MYLK3*, most prominently in totally debulked patients. Patients with any RD also appear to gain significant survival benefit by having higher methylation of *MYLK3.* The protective benefit of higher *MYLK3* methylation pulls the survival curve for the RD group into a similar position to the totally debulked patients with low methylation and appears to be a protective factor itself.

### Expression of *MYLK3* is not directly linked to survival or methylation of the *MYLK3* locus

**Figure 4:**
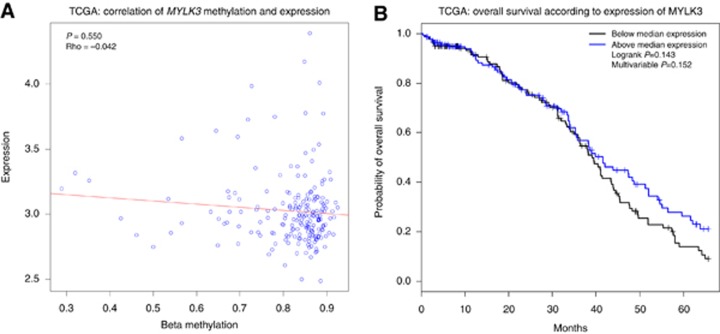
**Expression correlations with survival and methylation of *MYLK3*.** (**A**) Correlation plot of *MYLK3* expression and methylation showing no correlation (Pearson’s *ρ* −0.042, *P*=0.550) between the methylation level of *MYLK3* and expression of the gene. (**B**) Kaplan–Meier survival curve showing no significant relationship between *MYLK3* expression and OS (log-rank *P*=0.156, Cox *P*=0.079 (HR 0.70, 95% CI 0.47, 1.04)).

Hammersmith and TOC-Charité data sets did not have matched expression data, so TCGA expression data set was interrogated separately. All 277 patients in TCGA data set who had methylation data had matched expression data for *MYLK3*. Potential correlation between methylation and expression was investigated with Spearman’s rank correlation test (Figure 4A). There was no significant correlation between methylation of the *MYLK3* locus and expression of the *MYLK3* gene (*P*=0.55, *ρ*=−0.042). Furthermore, there was no statistically significant association between expression of the gene and OS (log-rank *P*=0.143, Cox *P*=0.152; Figure 4B). The same analysis was also performed on the other five discovery genes, all of which similarly showed no association with survival (data not shown).

## Discussion

Currently, all patients with advanced stage OC are treated with surgery and chemotherapy, despite our expanding knowledge of the heterogeneity of the disease. The benefit of achieving total macroscopic clearance of disease is now well recognised and these data show that this has served patients well in the past, as survival is significantly improved by performing increasingly radical surgery. However, although this blanket approach improves survival across a population, it takes no account of individual tumour biology and therefore is not necessarily the best treatment for all patients. Increasingly, more is known about the biology of tumours, chemotherapy evasion, the development of resistance, and the process of metastasis: future treatments will increasingly be focused on exploiting these unique characteristics. In the new age of personalised medicine, where patients should expect to have their care tailored for their individual needs, it is essential to determine biomarkers that will allow the stratification of care and to truly personalise medicine. Surgical biomarker studies tend to have poor surgical annotations, and most studies are limited by heterogeneous samples and lack adjustment for major confounding factors (Borley *et al*, 2012). Clinical, histological, and surgical annotations in this study were collected to a very high standard, mitigating some of the limitations inherent in previous surgical biomarker research.

*MYLK3* is a protein-coding gene found on chromosome 16 (16q11.2) recognised for its role in the regulation of actin cytoskeleton and immune response signalling. It phosphorylates cardiac myosin heavy (MYH7B) and light (MYL2) chains, potentiating the force and rate of cross bridge recruitment in myocytes (Chan *et al*, 2008). Also, myosin light chain kinases have been linked to the regulation of epithelial cell survival, and knockdown of the gene has led to apoptosis of epithelial breast line cells *in vitro* (Connell and Helfman, 2006). Myosin light chain kinase on chromosome 3, has a role in epithelial tight junction permeability, has been shown to be a warning marker for gastric cancer and has also been shown to promote cell proliferation (Al-Sadi *et al*, 2008; Han *et al*, 2011; Chen *et al*, 2012). It is not clear why differential methylation at this CpG locus in *MYLK3* seems to affect OS in patients who have received maximal surgical effort. Our findings may represent the OC cells’ ability to invade their basement membrane, move across epithelial junctions, and/or have a role in metastasis. To our knowledge there is no current evidence showing any association between DNA methylation of the *MYLK3* gene promoter region and expression of the gene. Nor is there any literature linking *MYLK3* to OC outcomes. Differential CpG island methylation is often associated with gene expression, but *MYLK3* does not contain a regulatory CpG island in its promoter region. Differential methylation can however affect a locus outside a CpG island by influencing transcription factor binding (Medvedeva *et al*, 2014). The locus examined here sits within the promoter region of the gene, only 45 base pairs from the transcription start site, but not as part of a CpG island. It is therefore not likely that methylation at the locus would have a direct effect on expression of *MYLK3* specifically. An alternative hypothesis is that methylation at this locus could be a surrogate marker for effects on the transcription of other genes. Further work should be performed to establish the exact nature, cause, and effect of this differential methylation on OC survival. In the meantime, differential methylation at the cg13247990 *MYLK3* locus appears to have significant potential as a surgical biomarker, which may enable stratification of surgical care according to the biology of patients’ tumours. The *MYLK3* biomarker could potentially be measured from tumour biopsy samples or from cell-free circulating tumour DNA (Bettegowda *et al*, 2014). Those patients with high methylation appear to have the best outcome from total macroscopic debulking and could therefore be assigned the most radical surgery. Women with low methylation may not benefit from radical upfront surgery, and other treatment strategies may be more beneficial, including the consideration of neoadjuvant chemotherapy. Clearly, the potential benefits of our findings would need to be confirmed in an appropriately controlled clinical trial.

In the Hammersmith database cohort of 430 women, 8.8% of totally debulked patients had progressed or died within 12 months of surgery, with very poor median OS of 3 months. It is questionable how much benefit these women received from radical surgery and our conventional treatment approaches. Differential methylation of *MYLK3* could potentially have an exciting part in the future of personalised medicine in OC surgery. The ability to determine which women will respond well to radical surgery would be a significant advancement in the treatment of OC.

## Figures and Tables

**Table 1:** Six most significant differentially methylated loci in Hammersmith discovery cohort using 27k methylation array associated with survival in patients with the least residual disease (optimal debulk *n*=39)

**Probe ID**	**Gene name**	**Median methylation %**	**Cox model (*****P***)^a^	**HR**	**95% CI**
cg14578030	*FGF4*	85.2	0.011	0.39	0.19, 0.81
cg21856603	*ITGAE*	77.5	0.042	0.48	0.24, 0.98
cg16155702	*FGF21*	74.3	0.031	0.50	0.26, 0.94
cg13247990	*MYLK3*	80.1	0.008	0.51	0.31, 0.84
cg19961522	*MYLK2*	73.2	0.024	0.57	0.35, 0.93
cg23370883	*MYL7*	61.7	0.026	0.57	0.35, 0.94

Abbreviations: CI=confidence interval; *FGF4*=fibroblast growth factor 4; *FGF21*=fibroblast growth factor 21; HR=hazards ratio; *ITGAE*=integrin alpha E; *MYLK2*=myosin light chain kinase 2; *MYLK3*=myosin light chain kinase 3; *MYL7*=myosin light chain 7, regulatory.

a *P*-value determined by Cox proportional hazards model adjusting for age, stage, chip, and residual disease status. Supplementary Table S3 lists the multivariable Cox proportional hazards survival analysis for the top 27 significant probes.

**Table 2:** Six candidate loci validation analysis in the Charité cohort using bisulphite pyrosequencing of differentially methylated loci associated with survival in patients with the least residual disease (total debulk *n*=47)

**Probe ID**	**Gene name**	**Median methylation %**	**Cox model (*****P***)^a^	**HR**	**95% CI**
cg14578030	*FGF4*	92.2	0.355	0.68	0.30, 1.55
cg21856603	*ITGAE*	NA	NA	NA	NA
cg16155702	*FGF21*	78.4	0.064	0.44	0.18, 1.05
cg13247990	*MYLK3*	86.3	0.053	0.51	0.21, 1.01
cg19961522	*MYLK2*	90.5	0.998	1.00	0.43, 2.30
cg23370883	*MYL7*	77.0	0.224	0.59	0.26, 1.38

Abbreviations: CI=confidence interval; *FGF4*=fibroblast growth factor 4; *FGF21*=fibroblast growth factor 21; HR=hazards ratio; *ITGAE*=integrin alpha E; *MYLK2*=myosin light chain kinase 2; *MYLK3*=myosin light chain kinase 3; *MYL7*=myosin light chain 7, regulatory; NA=not available.

*ITGAE* results not available as pyrosequencing assay failed.

a *P*-value determined by Cox proportional hazards model adjusting for age, stage, and grade.
