# Supplementary material for: Methylation of MYLK3 gene promoter region: a biomarker to stratify surgical care in ovarian cancer in a multicentre study
Source: Br J Cancer. 2017 Mar 28;116(10):1287–93. doi: 10.1038/bjc.2017.83 (PMC5482730; doi:10.1038/bjc.2017.83)
Supplement: Supplementary Table S1 [file bjc201783x1.docx]

Supplementary Table S1: Clinical characteristics of the historical Hammersmith database and the DNA methylation datasets used throughout the study

|  |  | **Hammersmith Database** | | **Hammersmith Dataset** | | **Charité Dataset** | | **TCGA Dataset** | |
| --- | --- | --- | --- | --- | --- | --- | --- | --- | --- |
|  |  | n= | 430 | n= | 70 | n= | 89 | n= | 277 |
| **Age mean (range)** | | 61·4y | (18-90) | 61·0y | (30-87) | 59·4y | (35-92) | 60.2y | (35-85) |
|  |  | Number | Percent (%) | Number | Percent (%) | Number | Percent (%) | Number | Percent (%) |
| **Stage** | 1&2 | 0 | 0·0 | 0 | 0·0 | 2 | 2·2 | 0 | 0·0 |
|  | 3 (NOS) | 9 | 2·1 | 0 | 0·0 | 0 | 0·0 | 0 | 0·0 |
|  | 3a | 18 | 4·2 | 0 | 0·0 | 1 | 1·1 | 3 | 1·1 |
|  | 3b | 32 | 7·4 | 5 | 7·1 | 2 | 2·2 | 14 | 5·1 |
|  | 3c | 247 | 57·4 | 47 | 67·1 | 61 | 68·5 | 212 | 76·5 |
|  | 4 | 124 | 28·8 | 18 | 25·7 | 21 | 23·6 | 48 | 17·3 |
|  | Unknown | 0 | 0·0 | 0 | 0·0 | 2 | 2·2 | 0 | 0·0 |
| **Grade** | 1 | 20 | 4·7 | 2 | 2·9 | 0 | 0·0 | 0 | 0·0 |
|  | 2 | 69 | 16·0 | 15 | 21·4 | 5 | 5·6 | 27 | 9·7 |
|  | 3 | 333 | 77·4 | 53 | 75·7 | 28 | 31·5 | 245 | 88·4 |
|  | Unknown | 8 | 1·9 | 0 | 0·0 | 56 | 62·9 | 5 | 1·8 |
| **Surgery** | Total debulk | 140 | 32·6 | 15 | 21·4 | 47 | 52·8 | 45 | 16·2 |
|  | Optimal debulk | 117 | 27·2 | 24 | 34·3 | 36 | 40·4 | 156 | 56·3 |
|  | Suboptimal debulk | 151 | 35·1 | 31 | 44·3 | 6 | 6·7 | 76 | 27·4 |
|  | Unknown | 22 | 5·1 | 0 | 0·0 | 0 | 0·0 | 0 | 0·0 |

(NOS: Not otherwise specified)
