# Supplementary material for: Methylation of MYLK3 gene promoter region: a biomarker to stratify surgical care in ovarian cancer in a multicentre study
Source: Br J Cancer. 2017 Mar 28;116(10):1287–93. doi: 10.1038/bjc.2017.83 (PMC5482730; doi:10.1038/bjc.2017.83)
Supplement: Supplementary Table S2 [file bjc201783x2.docx]

Supplementary Table S2:

DNA primer design for PCR and pyrosequencing.

| **Gene Name/ Probe ID** | **Primer** | | **Sequence** |
| --- | --- | --- | --- |
| FGF4 cg14578030 | Forward | 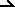 | AGGTGTAGAAAGGGGGGTATT |
|  | Reverse (bio) | 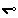 | AACCCACCTCCCATATCAT |
|  | Sequencing | 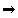 | TTGGTAGGATAGGAATTTTAGT |
| FGF21 cg16155702 | Forward | 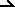 | AGTTTAGGGTGGGAGAGGT |
|  | Reverse (bio) | 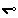 | TCACCCATCCATACAAAACCCCATCTAA |
|  | Sequencing | 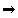 | TGTAGTTGAAAGTTTTGAAG |
| MYLK2 cg19961522 | Forward | 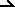 | GAGGGGAAATTGGATAGTTTGA |
|  | Reverse (bio) | 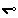 | AAACTCCTCCCTCCATCTC |
|  | Sequencing | 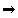 | AGTATAAGTTTTTTTATTTTATGG |
| MYLK3 cg13247990 | Forward | 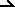 | TAGGGTGGGAGATAAGTAAAGT |
|  | Reverse (bio) | 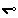 | AACTCTCCTTAAAAATTCCTAACATACT |
|  | Sequencing | 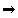 | GGAGGATAATGATTTTGTAGAT |
| MYL7 cg23370883 | Forward | 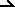 | AGGATGGGATTTGAGGTTATTG |
|  | Reverse (bio) | 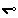 | CCCCTCCCTACTCACAATATTACAAA |
|  | Sequencing | 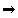 | GGTTTGTTTAAATATGGAAAAG |
| ITGAE cg21856603 | Forward | 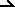 | ATATGTGTGGGGAGTGTTTAG |
|  | Reverse (bio) | 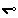 | CCCACTCTCAATCACTCAAATCTTAC |
|  | Sequencing | 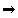 | GGGAGTGTTTAGGGTAA |

bio = biotinylated primer
