# Supplementary material for: Methylation of MYLK3 gene promoter region: a biomarker to stratify surgical care in ovarian cancer in a multicentre study
Source: Br J Cancer. 2017 Mar 28;116(10):1287–93. doi: 10.1038/bjc.2017.83 (PMC5482730; doi:10.1038/bjc.2017.83)
Supplement: Supplementary Table S3 [file bjc201783x3.docx]

Supplementary Table S3:

List of 27 significant differentially methylated loci associated with survival in the optimally debulked Hammersmith discovery dataset.

| **Probe ID** | **Gene Name** | **Cox model (*P=*)*** | **HR** | **95% C.I.** |
| --- | --- | --- | --- | --- |
| cg14578030 | FGF4 | 0.011 | 0.39 | (0.19, 0.81) |
| cg21856603 | ITGAE | 0.042 | 0.48 | (0.24, 0.98) |
| cg16155702 | FGF21 | 0.031 | 0.50 | (0.26, 0.94) |
| cg13247990 | MYLK3 | 0.008 | 0.51 | (0.31, 0.84) |
| cg19961522 | MYLK2 | 0.024 | 0.57 | (0.35, 0.93) |
| cg23370883 | MYL7 | 0.026 | 0.57 | (0.35, 0.94) |
| cg09528351 | PIK3R5 | 0.015 | 0.74 | (0.58, 0.94) |
| cg09517019 | ARHGEF6 | 0.014 | 0.75 | (0.59, 0.94) |
| cg17177660 | VEGFC | 0.002 | 0.75 | (0.62, 0.90) |
| cg08816023 | FGF1 | 0.017 | 0.77 | (0.62, 0.95) |
| cg21030598 | PAK2 | 0.048 | 0.79 | (0.63, 1.00) |
| cg14176836 | ITGAL | 0.037 | 0.80 | (0.65, 0.99) |
| cg05313261 | MAPK3 | 0.036 | 0.81 | (0.67, 0.99) |
| cg03543593 | TNXB | 0.012 | 0.82 | (0.70, 0.96) |
| cg04663194 | ACTN3 | 0.017 | 0.83 | (0.71, 0.97) |
| cg00833777 | ITGAM | 0.028 | 0.83 | (0.70, 0.98) |
| cg20795401 | ITGA6 | 0.037 | 0.85 | (0.73, 0.99) |
| cg03731616 | MYLK | 0.006 | 0.87 | (0.79, 0.96) |
| cg21902327 | FGF6 | 0.042 | 0.87 | (0.76, 1.00) |
| cg09538287 | CTNNA3 | 0.043 | 0.88 | (0.77, 1.00) |
| cg17694877 | DIAPH2 | 0.018 | 0.88 | (0.80, 0.98) |
| cg16154416 | RASGRF1 | 0.017 | 0.91 | (0.85, 0.98) |
| cg17286640 | KDR | 0.04 | 0.92 | (0.85, 1.00) |
| cg06958829 | CHAD | 0.043 | 1.09 | (1.00, 1.18) |
| cg14950072 | LAMA1 | 0.028 | 1.13 | (1.01, 1.26) |
| cg26571739 | VAV1 | 0.043 | 1.15 | (1.01, 1.31) |
| cg21671476 | MYL9 | 0.017 | 1.21 | (1.04, 1.42) |

Double line used to mark the top six loci.* *P* value determined by Cox proportional hazards model adjusting for age, stage, chip and residual disease status
