# Supplementary material for: Methylation of MYLK3 gene promoter region: a biomarker to stratify surgical care in ovarian cancer in a multicentre study
Source: Br J Cancer. 2017 Mar 28;116(10):1287–93. doi: 10.1038/bjc.2017.83 (PMC5482730; doi:10.1038/bjc.2017.83)
Supplement: Supplementary Table S4 [file bjc201783x4.docx]

Supplementary Table S4: TCGA dataset validation analysis of all six genes in the discovery set

| **Probe ID** | **Gene Name** | **Median methylation %** | **Logrank (*P*=)** | **Cox model (*P*=)*** | **HR** | **95% C.I.** |
| --- | --- | --- | --- | --- | --- | --- |
| cg14578030 | FGF4 | 87.1 | 0.863 | 0.826 | 1.04 | (0.71, 1.53) |
| cg21856603 | ITGAE | 77.3 | 0.554 | 0.417 | 0.85 | (0.59, 1.25) |
| cg16155702 | FGF21 | 86.5 | 0.869 | 0.996 | 1.00 | (0.68, 1.47) |
| cg13247990 | MYLK3 | 85.1 | 0.009 | 0.021 | 0.64 | (0.44, 0.93) |
| cg19961522 | MYLK2 | 77.4 | 0.555 | 0.686 | 0.93 | (0.64, 1.35) |
| cg23370883 | MYL7 | 64.1 | 0.240 | 0.093 | 1.39 | (0.95, 2.07) |

* Multivariable Cox model adjusted for age, stage, grade, debulk status
